# *Alternaria alternata* Accelerates Loss of Alveolar Macrophages and Promotes Lethal Influenza A Infection

**DOI:** 10.3390/v12090946

**Published:** 2020-08-27

**Authors:** Caroline M. Percopo, Michelle Ma, Eric Mai, Jamie L. Redes, Laura S. Kraemer, Mahnaz Minai, Ian N. Moore, Kirk M. Druey, Helene F. Rosenberg

**Affiliations:** 1 Inflammation Immunobiology Section, Laboratory of Allergic Diseases, National Institute of Allergy and Infectious Diseases, National Institutes of Health, Bethesda, MD 20892, USA; percopoc@niaid.nih.gov (C.M.P.); michelle.ma@nih.gov (M.M.); eric.mai96@gmail.com (E.M.); laura.s.kraemer@gmail.com (L.S.K.); 2 Lung and Vascular Inflammation Section, Laboratory of Allergic Diseases, National Institute of Allergy and Infectious Diseases, National Institutes of Health, Bethesda, MD 20892, USA; jamieredes@gmail.com (J.L.R.); kdruey@niaid.nih.gov (K.M.D.); 3 Infectious Disease Pathogenesis Section, Comparative Medicine Branch, National Institute of Allergy and Infectious Diseases, National Institutes of Health, Bethesda, MD 20892, USA; minaim@niaid.nih.gov (M.M.); ian.moore@nih.gov (I.N.M.)

**Keywords:** fungal rhinitis, cytokine, alveolar macrophage, *Alternaria alternata*, neutrophil, *Lactobacillus plantarum*

## Abstract

Chronic inhalation of fungi and fungal components has been linked to the development of respiratory disorders, although their role with respect to the pathogenesis of acute respiratory virus infection remains unclear. Here, we evaluate inflammatory pathology induced by repetitive administration of a filtrate of the ubiquitous fungus, *Alternaria alternata*, and its impact on susceptibility to infection with influenza A. We showed previously that *A. alternata* at the nasal mucosae resulted in increased susceptibility to an otherwise sublethal inoculum of influenza A in wild-type mice. Here we demonstrate that *A. alternata*-induced potentiation of influenza A infection was not dependent on fungal serine protease or ribonuclease activity. Repetitive challenge with *A. alternata* prior to virus infection resulted proinflammatory cytokines, neutrophil recruitment, and loss of alveolar macrophages to a degree that substantially exceeded that observed in response to influenza A infection alone. Concomitant administration of immunomodulatory *Lactobacillus plantarum*, a strategy shown previously to limit virus-induced inflammation in the airways, blocked the exaggerated lethal response. These observations promote an improved understanding of severe influenza infection with potential clinical relevance for individuals subjected to continuous exposure to molds and fungi.

## 1. Introduction

Influenza infection remains among the most important of the circulating respiratory virus pathogens. Although vaccination against influenza has been available since the mid-1940s, the U.S. Centers for Disease Control and Prevention reports that between 9 and 45 million individuals are infected with influenza each year, resulting in 12,000–61,000 deaths [1]. Similarly, while some individuals experience mild symptoms only, others can progress to severe pneumonia and acute respiratory distress syndrome (ARDS). While there are several known pre-existing conditions that are associated with severe disease [2], we do not have a complete understanding of the factors and mechanisms promoting virulent disease and influenza-associated mortality.

Fungi are ubiquitous in indoor and outdoor spaces [3]. Inhalation of fungi and/or fungal components has been linked to development of several disorders of the respiratory tract [4,5]. Specifically, exposure to spores from the saprophytic plant pathogen, *Alternaria alternata* (Family Pleosporaceae) has been associated with the development of asthma and allergic rhinitis, notably in warm and arid climates [6,7]. Sensitivity to *Alternaria* is prevalent in the adult population and has been reported to be a strong predictor of susceptibility to “thunderstorm”-related asthma [8].

*Alternaria* filtrates and extracts, both with and without adjuvant, have been used to model Th2 type inflammatory responses and airway hyperresponsiveness characteristic of allergic airways disease in mouse models [9]. Among these findings, Kita and colleagues [10,11] have explored the role of epithelial cytokines in promoting airways hyperreactivity in response to *A. alternata* allergens. Boitano and colleagues [12] demonstrated that the Th2-type responses to *A. alternata* were directly dependent on fungal serine protease activity; Yee and colleagues [13] identified and characterized an *Alternaria*-derived serine protease and documented inflammatory cell recruitment and airways dysfunction that results from its activation of protease-activated receptor 2 (PAR2).

By contrast, a role for *Alternaria* in promoting non-Th2 inflammatory pathology has not been explored as extensively. Among several recent findings, Babiceanu and colleagues [14] evaluated global gene expression in human bronchial epithelial cells challenged with *A. alternata* spores and reported differential expression of transcripts encoding proinflammatory cytokine and chemokine genes. Similarly, Daines and colleagues [15] showed that filtrates derived from *A. alternata* promote PAR2-independent synthesis of proinflammatory cytokines, including IL-6 and CXCL1, from primary mouse respiratory epithelial cells.

In a previous study, we developed and characterized an allergic rhinitis model featuring repetitive inoculation of minimal volumes of filtrate from *A. alternata* culture. This strategy led to eosinophilic inflammation in the nasal mucosa accompanied by local production of Th2-type and eosinophil chemoattractant cytokines, including IL-5, IL-13, and eotaxin-1 [16]. Previous findings revealed a role for eosinophils in promoting host defense against respiratory viruses [17,18,19], including Influenza A/H1N1 [20]. However, we found that eosinophils recruited to the nasal mucosa in response to *A. alternata* challenge had no impact on replication or clearance of Influenza A/H3N2 (Inf A) from the upper or lower airways. By contrast, we found that repetitive challenge with *A. alternata* led to an eosinophil-independent *increased* susceptibility to the lethal sequelae of Influenza A (Inf A) infection [16].

Here, we examine the inflammatory pathology elicited by *A. alternata* challenge and its impact on the susceptibility to infection with Inf A. In addition, we evaluated the impact of an anti-inflammatory/immunomodulatory strategy which suppresses the amplified lethal response to virus infection.

## 2. Materials and Methods

### 2.1. Mice

Wild-type female C57BL/6 mice (6–12 weeks old) were from the Jackson Laboratory, Bar Harbor, ME, USA (JAX 000664) and Charles River Laboratories, Frederick, MD, USA (stock 556). Age and gender-matched *PAR2*^−/−^ mice (JAX 004993) were from the Jackson Laboratory and maintained on site at the 14BS vivarium at NIAID/NIH, Bethesda, MD, USA. The National Institute of Allergy and Infectious Diseases Division of Intramural Research Animal Care and Use Committee, as part of the National Institutes of Health Intramural Research Program, approved all the experimental procedures as per protocol LAD 8E (approved 20 November 1998).

### 2.2. Fungal Filtrate Challenge and Inf A Infection

Mice under isoflurane anesthesia were inoculated intranasally with dilutions of a reconstituted filtrate of *A. alternata* (Stallergenes Greer, Lenoir, NC, USA; stock solutions at 10 mg/mL in Hanks’ buffered saline solution). Concentrations as indicated (0–50 μg/mouse) or diluent alone (sterile phosphate-buffered saline (PBS) with 0.1% bovine serum albumin (BSA)) were delivered in strict intranasal doses of 2.5 μL per nare [21] on days −19, −17, −15 (week 1), −12, −10, −8 (week 2), and −5, −3, and −1 (week 3). In some experiments, only three inoculations of *A. alternata* were performed prior to virus inoculation. In other experiments, the concentrated stock solutions of *A. alternata* (10 mg/mL) were subjected to dialysis against 500 volumes of sterile PBS to eliminate molecules smaller than 12 kDa (Spectra/Por, Spectrum Laboratories, Rancho Dominguez, CA, USA). Influenza A/HK/1/68 (gift from J. Keicher, Symmune Therapeutics, Raleigh, NC, USA) was provided to us as egg-passaged stock and was passaged three times in wild-type specific pathogen-free mice in our high-barrier facility in order to eliminate egg antigens prior to their utilization in in vivo experiments. We detected no increase in virus pathogenicity when comparing weight loss in response to passage 1 vs. passages 2 and 3. Influenza virus stocks were maintained at −80 °C as clarified lung homogenates in phosphate buffered saline (PBS) at 3 × 10^7^ TCID_50_ units/mL. *A. alternata* or diluent-challenged mice were inoculated under isoflurane anesthesia with Inf A at 30 tissue culture infectious dose (TCID)_50_ units/mouse in a total volume of 2.5 μL per nare on day 0; serial weights and survival were evaluated. Endpoints for mice in these studies included weight loss ≥25% from baseline and/or moribundity (lethargy and/or unwillingness to move when prompted). In other experiments, mice were inoculated as described and sacrificed on day 6 or day 8 for experimental readouts as indicated.

### 2.3. Virus Titration by qPCR

Virus was evaluated in bronchoalveolar (BAL) Fluid using a quantitative reverse transcription PCR (qPCR) assay that targeted the Inf A/HK/1/68 matrix (M1) protein as previously described [22]. Briefly, RNA was prepared from whole BAL fluid [14] and treated with DNase I to remove genomic DNA contaminants. Reverse transcription was performed using a first-strand cDNA synthesis kit (Roche, Nutley, NJ, USA) with random primers; a minus-reverse transcriptase control was included. The qPCR reactions were amplified in triplicate using ABI 2× TaqMan reagent, primer-probe mixes, and cDNA or plasmid standard in a 25 μL final volume (Applied Biosystems, Foster City, CA, USA). Thermal cycling parameters for the ABI7500 absolute quantitation program (Applied Biosystems, Foster City, CA, USA) include 50 °C for 2 min, 95 °C for 10 min, and 40 amplification cycles alternating 95 °C for 15 s and 60 °C for 1 min. Custom design primer-probes include primer 1, 5′-AAG ACC AAT CCT GTC ACC-3′; primer 2, 5′-CAA AGC GTC TAC GCT GCA GTC C-3′; and probe 6FAM-TTT GTG TTC ACG CTC ACC GTG CC-TAMRA). A 1002 bp PCR amplicon of the M1 protein (bp 1–1002, GenBank ID CY112250.1) was used to generate a standard curve for absolute quantification. Experimental triplicate data points were interpolated to linear standard curves over the concentration ranges indicated. Virus titration data from BAL fluid are presented as copies/µL.

### 2.4. Lung Wet-to-Dry Measurements

Inflammation and associated pulmonary edema were evaluated quantitatively as previously described [23,24]. Briefly, whole lungs were removed from Inf A-infected and *A. alternata*-challenged and Inf A-infected mice. Lung wet weights and dry weights (significant to 0.05 g, Denver Instruments top-loading balance, Model SI-403, Arvada, CA, USA) were determined immediately after removal (wet) and again after evaporation in a drying oven at 37 °C for 7 days (dry). The ratio of the wet lung weight to the dry lung weight at each time point was determined.

### 2.5. Lung Histopathology and Immunohistochemistry

Lung tissues were perfused with 10 mL PBS prior to removal from the body cavity, fixed in 10% neutral buffered formalin, sectioned, and blocked in paraffin for histological analysis. Tissue sections (5 μm) were stained with hematoxylin and eosin (H&E) for routine histopathology. Immunohistochemical detection of Influenza-NP antigen was performed using rabbit polyclonal anti-Influenza A NP (GeneTex, Irvine, CA, USA, Cat no. GTX125989) at a dilution of 1:4000. Staining was carried out on a Leica BOND RX Automated Stainer platform (Leica Biosystems, Buffalo Grove, IL, USA) according to manufacturer-supplied protocols. Briefly, 5-μm thick sections were baked, deparaffinized, and rehydrated. Heat-induced epitope retrieval was performed using Leica Epitope Retrieval Solution 1 (pH 6), heated to 100 °C for 20 min. The slides were then incubated with hydrogen peroxide to quench endogenous peroxidase activity prior to applying the aforementioned primary Ab. The secondary antibody was an anti-rabbit Poly-HRP-IgG which is a component of the Bond Polymer Refine Detection kit (Leica Biosystems, Buffalo Grove, IL, USA, Cat. no. DS9800). Diaminobenzidine chromogen detection was completed using the Bond Polymer Refine Detection kit and counterstained using hematoxylin (Leica Biosystems, Buffalo Grove, IL, USA). Slides were then cleared through gradient alcohol and xylene washes prior to mounting and placing cover slips. Sections were examined by a board-certified veterinary pathologist (INM) using an Olympus BX51 light microscope. Photomicrographs were taken using an Olympus DP73 camera.

### 2.6. Immunoreactive Cytokines in BAL Fluid

BAL fluid (1.6–1.8 mL in sterile PBS with 0.1% BSA) was collected from mice treated *A. alternata* or diluent alone followed by inoculation with Inf A or diluent as described above. Quantitative analysis of cytokine mediators was performed by ELISA (DuoSet, Quantikine; R&D Systems, Minneapolis, MN, USA) as per manufacturers’ instructions.

### 2.7. Flow Cytometry

For preparation of single-cell suspensions, all mice were euthanized by isoflurane inhalation followed by cervical dislocation at time points indicated. Peripheral blood was removed by perfusion with 8 mL of PBS with 10 mM EDTA; lungs were removed from the body cavity and minced followed by incubation in digestion buffer (RPMI 1640 with 5% FBS, 20 μg/mL DNase I, and 40 μg/mL collagenase D) for 90 min at 37 °C; tissue digests were filtered through 70-μm nylon mesh, and erythrocytes were lysed with ACK buffer (Lonza, Morristown, NJ, USA). Cells were counted on a hemocytometer and the fraction of live cells was identified by trypan blue exclusion (Lonza, Morristown, NJ, USA). For evaluation of myeloid subsets, cells (10^6^ cells per 100 μL) were stained with LIVE/DEAD Aqua or Blue (20 min at 4 °C; Invitrogen, Carlsbad, CA, USA) followed by anti-CD16/32 (BD Biosciences, San Jose, CA, USA) and fluorochrome-conjugated Abs, including: from eBioscience (San Diego, CA, USA): anti-CD45 (clone 30-F11), anti-CD11c (clone N418), anti–MHC class II (MHC II)–1-A/1-K (clone M5/114.15.2); from BD Biosciences (San Jose, CA, USA): anti–Siglec F (clone E50-2440), anti-Gr1 (clone RB6-8C5). For evaluation of T lymphocytes, single-cell suspensions generated as described above were stained with LIVE/DEAD Aqua (Invitrogen, Carlsbad, CA, USA) followed by anti-CD16/32, anti-CD45 (clone 30-F11), anti-CD19 (clone 1D3), anti-CD3 (clone eBIO500A2), anti-CD4 (clone RM4-5), and anti-CD8a (clone 53-6.7). Ab staining was performed for 20 min at 4 °C. Cell suspensions were then fixed in 4% paraformaldehyde in PBS prior to analysis on an LSR II flow cytometer (BD Biosciences, San Jose, CA, USA). For all analyses, compensation and analysis of flow cytometry data were performed using FlowJo 10.2 software (Tree Star, Ashland, OR, USA). Gates were set using relevant “fluorescence minus one” and isotype controls. Representative flow plots are shown Supplementary Figure S1.

### 2.8. Mouse Tracheal Epithelial Cell Cultures

Isolated mouse tracheal epithelial cells from C57BL/6 mice were generated and cultured as described [25]. Tracheal epithelial cells were added to the apical chamber of collagen-coated (rat tail type I, 50 µg/mL) polycarbonate 24-well trans-well plates (Costar, Washington, DC, USA); 0.5 mL mTEC/Plus medium (mTEC basic medium (DMEM/Hams F-12 medium, GIBCO), 15 mM HEPES, 4 mM glutamine, 3.6 mM NaHCO_3_, 5% FBS, penicillin/streptomycin and amphotericin B [0.25 µg/mL; GIBCO, Gaithersburg, MD, USA]) with 10 µg/mL insulin (Sigma-Aldrich, St. Louis, MO, USA), 5 µg/mL transferrin (Sigma-Aldrich, St. Louis, MO, USA), 0.1 µg/mL cholera toxin, (Sigma-Aldrich), 25 ng/mL Epidermal growth factor (BD Biosciences, San Jose, CA, USA), bovine pituitary extract (30 µg/mL; PelFreez, Rogers, AR, USA) and 50 nM retinoic acid (RA) was added to the basal chamber immediately prior to adding cells to the apical chamber. Resistance measurements performed with a volt/ohm meter (EVOM, World Precision Instruments, Sarasota, FL, USA) were used to evaluate confluence; once resistance of >1000 Ωcm^2^ was reached, the air–liquid interface was initiated. Medium from both apical and basal chambers was removed; the basal medium was replaced with mTEC/NuSerum (mTEC basic medium supplemented with 2% NuSerum (BD Biosciences, San Jose, CA, USA) and 0.5 mM retinoic acid. Warmed PBS was added to the cells in the apical chamber and immediately removed. Cilia appear after 3–4 days later and gradually increase in number. The apical surfaces of cultures maintained under these conditions for 5 days were challenged with 150 µL of 1 µg/mL dilution of *A. alternata* filtrate (Stallergenes Greer, Lenoir, NC, USA) or PBS diluent alone. Cells challenged with *A. alternata* were incubated for 2 h at 37 °C in 5% CO_2_; the apical chamber fluid was then aspirated, rinsed once with warmed PBS, and the cultures were returned to 32 °C, 5% CO_2_. This was repeated every two days for a total of three challenges. Two days after the final challenge with *A. alternata*, 150 µL of a 1:1000 dilution of mouse-adapted Inf A (Inf A/FM/1/47-MA [26]; gift Dr. S. L. Epstein, US Food and Drug Administration with permission from Dr. E. G. Brown, University of Ottawa) was added to the apical surface. The transwell plates were incubated 4 h at 32 °C in 5% CO_2_. The apical fluid was aspirated, washed with warmed PBS, and the cultures were returned to 32 °C, 5% CO_2_. At *t* = 4 days, basal medium was collected for ELISA and epithelial cells were collected for preparation of RNA for determination of virus titer. For preparation of RNA, RNA-bee was added to apical surface of mTEC cultures; RNA was prepared by standard methods and treated with DNase I. The M1 primers/probe specific for this virus strain include: Forward Primer 5′-GCGAGGACTGCAGCGTAGAC-3′; Reverse Primer 5′-GGATCCCCGTTCCCATTAAG-3′ and Probe 6FAM-CTTTGTCCAAAATGCMGBNFQ. A 594 bp PCR amplicon of the M1 protein (GenBank ID KY348534.1) cloned into the PCR2.1 vector was used to generate a standard curve for absolute quantification as described above. These values were normalized to absolute copies of GAPDH that were generated using commercially available mouse GAPDH primer-probes (Applied Biosystems, Foster City, CA, USA catalog no. 4308313); values obtained were interpolated to a standard curve generated using a mouse GAPDH plasmid in pCMV pSport 6 (American Type Culture Collection, Manassas, VA, USA, Cat. no. 10539385), also as previously described [22].

### 2.9. Protease Activity Assays

Protease activities of native and 4-(2-aminoethyl) benzenesulfonyl fluoride hydrochloride (AEBSF)-treated filtrates of *A. alternata* were assessed using the FluoroProtease Assay kit (G-Biosciences, St. Louis, MO, USA) as previously described [16]. This assay uses fluorescein isothiocyanate (FITC)-labeled casein as a pan-protease substrate. Samples were diluted in 1× Fluoro^™^ Assay Buffer and added to wells of a 96-well fluorometer-compatible titer plate. FITC-conjugated casein assay substrate was added to the wells and incubated at room temperature for 2 h. Fluorescence intensity was determined using a FilterMax F5 multi-mode microplate reader at an excitation wavelength of 485 nm and an emission wavelength of 530 nm. Buffer without protease was used as a blank for background subtraction. AEBSF (200 mM stock solution in DMSO, Sigma Aldrich, St. Louis, MO, USA) was added to *A. alternata* extracts (10 mg/mL) at concentrations indicated (from 0 to 30 mM). After 2 h incubation at room temperature, reactions were dialyzed against cold PBS.

### 2.10. Ribonuclease Activity Assays

In total, 1 µL of fresh diethyl-pyrocarbonate (DEPC) stock solution was added to 600 µL *A. alternata* filtrate in a fume hood; 1 µL of dH_2_O was added to a second 600 µL aliquot of *A. alternata* filtrate as a control. Each sample was rotated end/end at room temperature for 6 h then dialyzed overnight at 4 °C against multiple changes of cold PBS using 6000 MW cut-off dialysis membrane (Spectrum Industries, Grand Rapids, MI, USA). Ribonuclease activities of native and DEPC-treated filtrates were assessed by standard ribonuclease assay as previously described with some minor modifications [27]. Briefly, to initiate a reaction, 10 μL of a 20 mg/mL solution of acid-insoluble yeast tRNA (Sigma-Aldrich, St. Louis, MO, USA) was added to 0.8 mL of 40 mM sodium phosphate, pH 7.5, containing 5 μL of native or DEPC-treated *A. alternata* extract. At *t* = 1 h, the reaction was stopped by addition of 0.5 mL of a fresh-prepared, ice-cold solution of 20 mM lanthanum nitrate with 3% perchloric acid. For the *t* = 0 control, the stop solution was added to *A. alternata* in phosphate buffer prior to the addition of the yeast tRNA. Stopped reactions were held on ice for at least 15 min, and the acid-insoluble tRNA was removed by centrifugation for 5 min at 10,000× *g*. Solubilized tRNA was determined as UV absorbance (OD_260_) measured in the supernatants, with the *t* = 0 control used as the blank. All points shown are an average of triplicate samples.

### 2.11. Bacterial Colony Forming Units (CFU)

**Figure 1:**
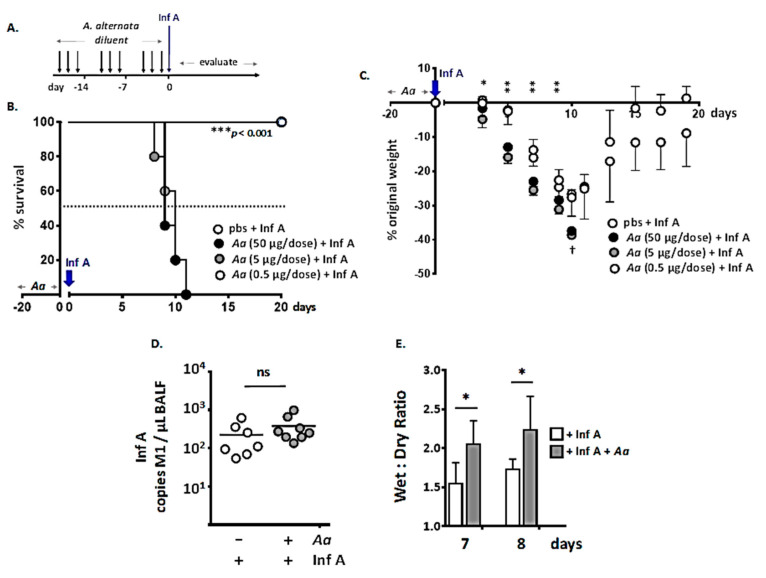
Repetitive administration of *Alternaria alternata* results in increased susceptibility to lethal Inf A infection. (**A**) Strategy: *A. alternata* at the nasal mucosa (0–50 µg per inoculum; 2.5 µL per nare) on days indicated followed by Influenza A/H3N2 (Inf A; 30 tissue culture infectious dose (TCID)_50_ units per mouse, 2.5 µL per nare) on day 0. (**B**) Survival in response to *A. alternata* followed by Inf A, as per the inoculation strategy in A.; *n* = 5 mice per group, *** *p* < 0.001, Kaplan–Meier log-rank. (**C**) Weight loss in response to *A. alternata* followed by Inf A as per the inoculation strategy in A.; *n* = 5 mice per group, * *p* < 0.05, ** *p* < 0.01, 1-way ANOVA; ^†^ no survival after this time point in response to the two highest doses of *A. alternata* as indicated. (**D**) Virus (copies virus M1 gene/µL bronchoalveolar lavage fluid [BAL]) determined by quantitative reverse transcription-polymerase chain reaction (qRT-PCR) on day 6 as per Figure 1A in mice treated with *A. alternata* (5 µg per inoculum) or diluent only prior to inoculation with Inf A; ns = no significant difference. (**E**) Wet-to-dry lung weight ratios at time points shown as per Figure 1A; 5 µg *A. alternata* per inoculum, *n* = 5 mice per group, * *p* < 0.05, 1-way ANOVA.

BAL fluid (1.6–1.8 mL in sterile PBS with 0.1% BSA) was collected from mice treated with *A. alternata* or diluent alone followed by Inf A or diluent as shown in Figure 1A; one cohort of mice in this experiment was also treated with ampicillin (20 mg/mouse/day via the intraperitoneal (i.p.) route) or diluent control. Serial 10-fold dilutions of 50 µL of BAL fluid were streaked on a blood agar plates (Thermo Fisher Scientific, Waltham, MA, USA) which were incubated overnight at 37 °C. Colonies were counted by visual inspection and were evaluated by Gram-stain followed by automated identification using the bioMérieux Vitek 2 System (Hazelwood, MO, USA).

### 2.12. Administration of Lactobacillus plantarum to the Respiratory Tract

*Lactobacillus plantarum* NCIMB 8826 (BAA-793; American Type Culture Collection, Manassas, VA, USA) was grown overnight in De Man Rogosa Sharp (MRS) medium, heat-inactivated (70 °C for 30 min), washed once in sterile PBS and stored at 10^11^/mL (see conversion from OD to cells/mL in [28]) in sterile PBS with 0.1% BSA at −20 °C. *L. plantarum* (10^8^ cells in 50 µL PBS with 0.1% BSA or diluent control) was administered to the respiratory tract just prior to *A. alternata* (5 µg/mouse in 2.5 µL per nare) on days −5, −3, and −1 followed by Inf A (30 TCID_50_ units per mouse, 2.5 µL per nare). One set of mice (*L. plantarum* and diluent control treated) were weighed on day 0 and again on day 5 and onwards. Other mice were euthanized on day 8 for cellular and biochemical evaluation as described above.

### 2.13. Statistical Evaluation

Data were evaluated with one-way analysis of variance (ANOVA), Kaplan–Meier log-rank or Mann–Whitney U-tests as appropriate; *p* values < 0.05 were considered to be significant.

## 3. Results and Discussion

### 3.1. Repetitive Administration of A. alternata Increases Susceptibility to Lethal Inf A Infection

In an earlier study [16], we found that repetitive administration of strict intranasal doses of a filtrate of *A. alternata* elicited profound eosinophil infiltration in the upper respiratory tract. This result was anticipated, as *A. alternata* is a prominent environmental allergen and has been used successfully to model respiratory allergy in mouse models [9,10,11,29]. However, despite earlier studies documenting antiviral properties of eosinophils in the airways [17,18,19], we found that repetitive administration of *A. alternata* resulted in an *increased* rather than decreased susceptibility to the lethal sequelae of a subsequent Inf A infection.

We explored these findings further here using the inoculation strategy shown in Figure 1A. Specifically, we confirmed that mice subjected to repetitive intranasal dosing with *A. alternata* had exaggerated responses with respect to the morbidity and mortality associated with Inf A infection; here, we show that the degree of this response was directly related to the dose of *A. alternata* (Figure 1B). Mice succumbed to an otherwise sublethal inoculum of Inf A after repetitive administration of *A. alternata* at 5 or 50 µg per dose; by contrast, all Inf A-infected mice survived when *A. alternata* was reduced to 0.5 µg per dose. No weight loss was observed in response to repetitive administration of *A. alternata* alone (0.5–50 μg/dose). Moreover, as shown in Figure 1C, all mice inoculated with diluent alone lost weight in response to Inf A infection but ultimately recovered completely by day 19; recovery was delayed among mice that underwent repetitive inoculation with *A. alternata* at 0.5 ug per dose prior to Inf A infection, although the difference from diluent alone did not reach statistical significance. Interestingly, administration of *A. alternata* had no impact on virus titers in the airways at day 6 of infection; this point was chosen because it is just prior to the onset of mortality in the cohort of mice inoculated with *A. alternata* (Figure 1D). However, lung wet-to-dry ratios, an indirect measure of inflammation and edema [23,24], were significantly higher in lungs of *A. alternata*-challenged Inf A-infected mice than in mice infected with Inf A alone (Figure 1E).

### 3.2. Repetitive Administration of A. alternata Amplifies the Biochemical Inflammatory Response to Infection with Inf A

**Figure 2:**
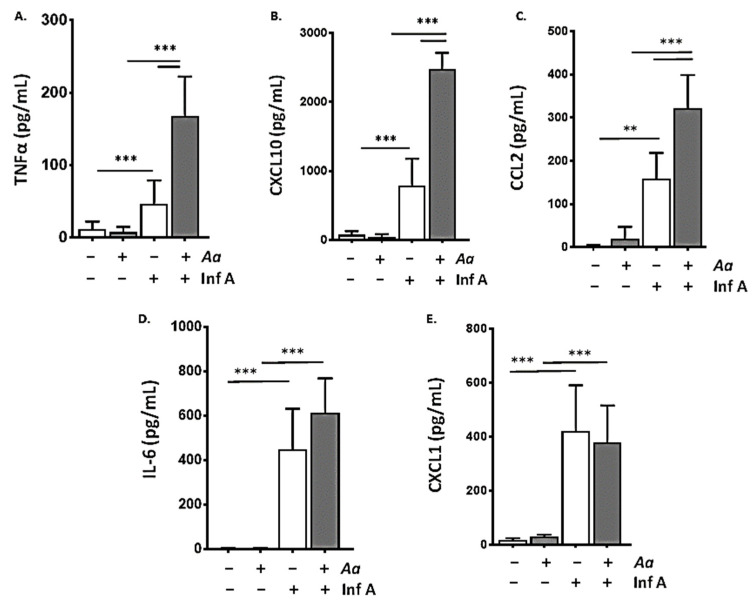
Repetitive administration of *A. alternata* amplifies the biochemical inflammatory response to infection with Inf A. Repetitive administration of *A. alternata* (5 µg per inoculum) as in Figure 1A results in amplification of (**A**) TNFα, (**B**) CXCL10, and (**C**) CCL2 in BAL fluid (day 6 as per Figure 1A) compared to responses observed in response to a sublethal inoculum of Inf A infection alone. By contrast, repetitive administration of *A. alternata* had no impact on levels of (**D**) IL-6 or (**E**) CXCL1 (day 6 as per Figure 1A) compared to those generated in response to Inf A infection alone. For all cytokines evaluated (a–e), administration of *A. alternata* alone (i.e., no Inf A) resulted in no detection over background levels; *n* = 5–8 mice per point, ** *p* < 0.01, *** *p* < 0.005, 1-way ANOVA.

Consistent with the findings shown in Figure 1E and our earlier studies that were focused on the inflammatory responses detected in nasal passages [16], we found that repetitive administration of *A. alternata* via strict intranasal doses as shown in Figure 1A resulted in amplified biochemical responses to Inf A in the lower airways. Links between inflammatory responses in the upper and lower airways have been considered extensively in the literature, notably with respect to clinical associations between allergic rhinosinusitis and asthma [30,31,32,33,34]. Several mechanisms have been proposed to account for these observations, including direct transfer of inciting agents and inflammatory mediators from the nasopharynx to the lower airways via fluid secretions and/or induction of systemic inflammation and transfer of proinflammatory mediators from the nasopharynx to the lung via peripheral circulation. As shown in Figure 2, inoculation with Inf A resulted in the production and release of proinflammatory cytokines in the lower airways (BAL fluid; ** *p* < 0.01; *** *p* < 0.005). Prior repetitive inoculation with strict intranasal volumes of *A. alternata* resulted in profound amplification of these cytokine responses. Among these, levels of TNFα, CXCL10, and CCL2 detected in BAL fluid in response to administration of *A. alternata* followed by Inf A exceeded those detected in response to Inf A alone (*** *p* < 0.005).

Inflammation observed in response to repetitive inoculation with *A. alternata* followed by Inf A infection is featured in Supplementary Figure S2. The enlargement shown in panel B highlights the inflammatory cells and necrotic cellular debris within larger airways, typical of severe influenza virus infection. Immunohistochemical staining for Inf A virus (Supplementary Figure S2C,D) documents virus NP protein in the bronchial epithelium and within inflammatory cells in the interstitial tissue, also consistent with a severe infection.

Previous reports suggested that airway epithelial cells may be critical targets of *A. alternata* in the respiratory tract. Notably, Babiceanu and colleagues [14] found that human epithelial BEAS-B cells responded to *A. alternata* challenge by producing proinflammatory cytokines, among them CXCL1, CXCL10, and CCL2. Interestingly, isolated mouse tracheal epithelial cells (mTECs [25]) produce and release proinflammatory cytokines in response to infection with Inf A; however, no amplification of this response was observed in response to ex vivo administration of *A. alternata* (Supplementary Figure S3).

### 3.3. Repetitive Administration of A. alternata Accelerates Depletion of Alveolar Macrophages in Response to Infection with Inf A

Alveolar macrophages (AMs) are primary sentinels that serve to promote host defense against infection with Inf A (reviewed in [35]). Ghoneim and colleagues [36] were among the first to report profound depletion of AMs in Inf A-infected BALB/c mice. Likewise, mice subjected to exogenous AM depletion [37] or those with defective AMs due to granulocyte macrophage-colony stimulating factor (GM-CSF) gene-deletion [38] were highly susceptible to the lethal sequelae of Inf A infection; Huang and colleagues [39] found that supplementation with GM-CSF supported AM survival and conferred resistance to lethal virus infection. Most recently, Magnen and colleagues [40] identified the host serine protease, kallikrein-1, as an endogenous factor that targets AMs and induces apoptosis response to acute influenza infection.

**Figure 3:**
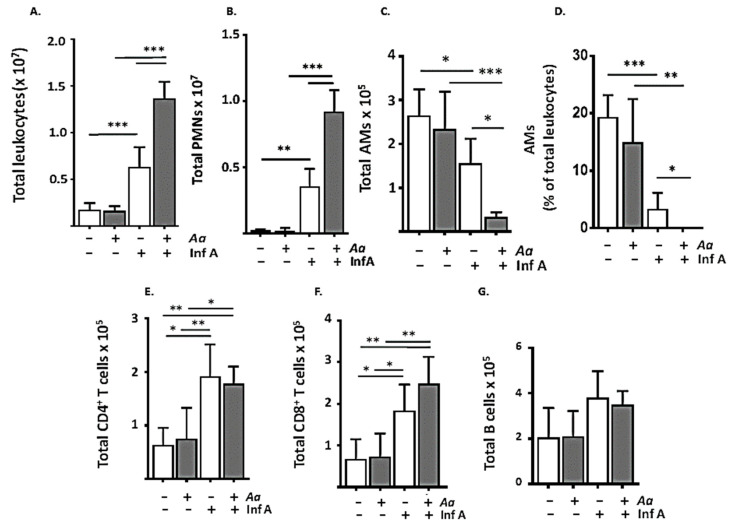
Repetitive administration of *A. alternata* amplifies total neutrophil recruitment and accelerates loss of alveolar macrophages in response to infection with Inf A. (**A**) Total leukocytes and (**B**) total PMNs recruited to lung tissue in mice treated with *A. alternata* (5 µg per inoculum) prior to infection with a sublethal inoculum of Inf A compared to that observed in response to inoculation with Inf A alone. (**C**) Infection with Inf A results in a loss of AMs; repetitive exposure to *A. alternata* (5 µg per inoculum) prior to Inf A infection amplifies this response. (**D**) AMs as percent of total leukocytes in *A. alternata*-treated Inf A-infected mice are reduced to undetectable levels; *n* = 5 per group, * *p* < 0.05, ** *p* < 0.01; *** *p* < 0.005, 1-way ANOVA. By contrast, repetitive administration of *A. alternata* (5 µg per inoculum) had no impact on (**E**) total CD4^+^ T cells, (**F**) total CD8^+^ T cells, or (**G**) total B cells recruited to the lungs in response to infection with Inf A; *n* = 5 mice per group, day 6 as per Figure 1A; * *p* < 0.05, ** *p* < 0.01, 1-way ANOVA.

To explore this issue in mice challenged with *A. alternata*, we performed a quantitative evaluation of total lung leukocytes and individual leukocyte lineages by flow cytometry (see Supplementary Figure S1). As shown in Figure 3A, Inf A infection alone resulted in a ≈3–4-fold increase in total lung leukocytes (0.17 ± 0.07 × 10^7^ vs. 0.63 ± 2.1 × 10^7^ cells, *** *p* < 0.005); this was amplified > 2-fold, to 1.4 ± 0.18 × 10^7^ cells in mice subjected to repetitive inoculation with *A. alternata* prior to Inf A infection (*** *p* < 0.005). Most of the increase was due to the influx of neutrophils (PMNs; Figure 3B) recruited both response to Inf A alone (to 0.36 ± 0.13 × 10^7^ cells) and at amplified levels in mice subjected to repetitive inoculation with *A. alternata* prior to infection with Inf A (to 0.92 ± 0.16 × 10^7^ cells; *** *p* < 0.005); neutrophils ultimately represented 68 ± 4% of the total leukocytes in lungs of *A. alternata*-challenged Inf A-infected mice.

The alveolar macrophage (AM) population also responded to both Inf A infection alone and Inf A infection after administration of *A. alternata*. As shown in Figure 3C, the total AMs were reduced from 2.7 ± 0.60 × 10^5^ in naïve mice to 1.6 ± 0.55 × 10^5^ cells in response to Inf A infection alone (* *p* < 0.05). However, Inf A infection preceded by repetitive inoculation with *A. alternata* resulted in a more extensive AM depletion; the combination of *A. alternata* and Inf A resulted in an additional 5-fold reduction in total AMs, to 0.34 ± 0.11 × 10^5^ cells (* *p* < 0.05). As shown, administration of *A. alternata* alone had no impact on the AM population. As a percent of total leukocytes, the fraction of AMs decreased from 19 ± 4% at homeostasis to 3.4 ± 2.8% in response to Inf A infection (*** *p* < 0.005; Figure 3D); AMs were virtually undetectable in Inf A-infected mice that undergone repetitive inoculation with *A. alternata*. By contrast, administration of *A. alternata* had no impact on the virus-induced recruitment CD4^+^ T cells (Figure 3E), CD8^+^ T cells (Figure 3F), or B cells (Figure 3G).

As considered further in sections to follow, small molecules derived from *A. alternata* may have a profound impact on the viability of both mouse and human macrophages. Among these findings, Solhaug and colleagues [41,42] reported that alternariol, a toxic dibenzopyrone metabolite of *Alternaria* species, promoted DNA damage, diminished phagocytic activity, and an altered the proinflammatory phenotype of the RAW264.7 mouse macrophage cell line. Interestingly, we found that AMs isolated from mice treated with *A. alternata* as in Figure 1A were fully viable in tissue culture and remained so for 5 days after infection with Inf A ex vivo.

### 3.4. Spontaneous Accumulation of Bacteria in the Airways of A. alternata-Challenged Inf A-Infected Mice

Given the observation that repetitive administration of *A. alternata* results in accelerated loss of AMs, we explored the possibility that the amplified lethal response might be related to diminished bacterial clearance from the respiratory tract.

There are numerous reports of increased susceptibility to acute bacterial infection as a complication or co-morbidity of influenza infection [43,44]. Most recently, Jochems and colleagues [45] examined this issue in human subjects challenged with live attenuated influenza virus and *Streptococcus pneumoniae* (type 6B); this study also demonstrated a correlation between bacterial load and levels of the proinflammatory cytokine CXCL10. Lethal synergism has also been observed in mouse studies, all of which include exogenous challenge with known bacterial pathogens [36,46,47,48,49,50].

**Figure 4:**
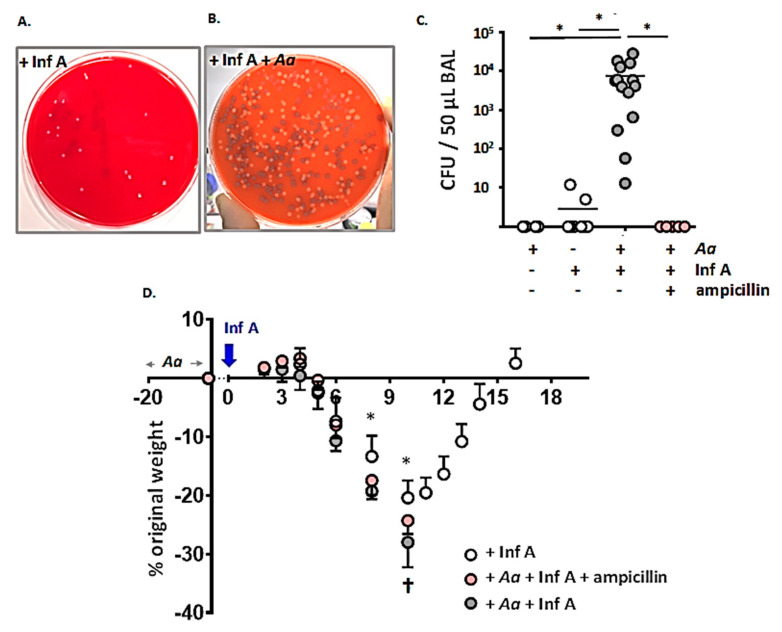
Bacterial colonization of the airways of *A. alternata*-challenged, Inf A-infected mice. Overnight growth at 37 °C on blood agar plates streaked with dilutions of BAL fluid from (**A**) a mouse infected with a sublethal inoculum of Inf A only and (**B**) a mouse subjected to repetitive inoculation with *A. alternata* (5 µg per inoculum) prior to Inf A infection. Both samples were from day 8 as per the timeline shown in Figure 1A. (**C**) Colony forming units (CFUs) per 50 µL BAL fluid on day 8 as per the timeline in Figure 1A. Shown are results from mice challenged with *A. alternata* alone, sublethal Inf A alone, *A. alternata* followed by Inf A, and *A. alternata* followed by Inf A and ampicillin (20 mg/mouse/day i.p. on days 0–8.) (**D**) Total body weight over time for mice in groups indicated in (C); mice treated with *A. alternata* alone (5 µg per inoculum) do not lose weight; *n* = 10 mice per group, * *p* < 0.05, 1-way ANOVA; ^†^ no survival after this time point in groups indicated.

Images of typical mixed bacterial growth on blood agar from BAL fluid samples from mice infected with Inf A alone and mice challenged with *A. alternata* prior to infection with Inf A are shown in Figure 4A,B. Few to no bacterial colonies were detected in BAL fluid from mice subjected to repetitive challenge with *A. alternata* alone or from mice infected with Inf A alone (Figure 4C); likewise, the concentrated stock solutions of *A. alternata* (10 mg/mL) and Inf A (3 × 10^7^ TCID_50_/mL) generated no colony forming units (CFUs) on blood agar plates. By contrast, we detected 7.4 ± 8.3 × 10^3^ CFU/50 µL BAL fluid from mice subjected to repetitive inoculation with *A. alternata* followed by infection with Inf A; this included spontaneous accumulation of both Gram-positive (*Staphylococcus* and *Streptococcus* spp.) and Gram-negative (*Escherichia coli*) microorganisms; all were identified as ampicillin-sensitive. As such, mice that were challenged with *A. alternata* followed by infection Inf A were also treated with ampicillin (20 mg/mouse/day i.p.) starting on day of Inf A inoculation (day 0) through day 8 thereafter; as shown in Figure 4C, no CFUs were detected in BAL fluid from mice from the ampicillin-treated cohort.

Given these observations, weight loss and survival in response to ampicillin treatment were evaluated as shown in Figure 4D. Interestingly, although no bacteria were detected in the airways of ampicillin-treated mice, this intervention had no impact on weight loss or survival in *A. alternata*-challenged, Inf A-infected mice.

The literature on bacterial infections that complicate influenza has largely focused on the Gram-positive pathogens, *S. pneumoniae* and *S. aureus* [43]; however, we cannot rule out the possibility that repetitive exposure to *A. alternata* and concomitant loss of AMs results in unrelated spontaneous and ampicillin-resistant bacterial infections that were not revealed by this methodology. In this case, quantitative analysis of bacterial 16S rRNA genes in BAL fluid or lung tissue (i.e., from mice that were challenged with *A. alternata*, Inf A, and/or ampicillin) might be revealing [51].

### 3.5. Administration of Immunobiotic Lactobacillus plantarum Directly to the Respiratory Mucosa Protects against the Lethal Impact of A. alternata

We and others have examined the impact of *L. plantarum* at the respiratory tract and its role in preventing a virus-induced inflammatory response [28,52,53,54]. Specifically, we found that live or heat-inactivated *L. plantarum* (BAA-793) administered directly to the respiratory tract had little to no impact on virus replication [28] but resulted in dramatically diminished production and release of critical virus-induced proinflammatory cytokines [28,55]. Administration of *L. plantarum* in this fashion ultimately resulted in full and sustained survival in response to an otherwise lethal respiratory virus infection. This response was not B or T cell dependent nor did it require IL-10, type I or type II interferon-mediated signaling [55,56,57]; however, maximum impact involved coordinate signaling via pattern recognition receptors TLR2 and NOD2 [58].

**Figure 5:**
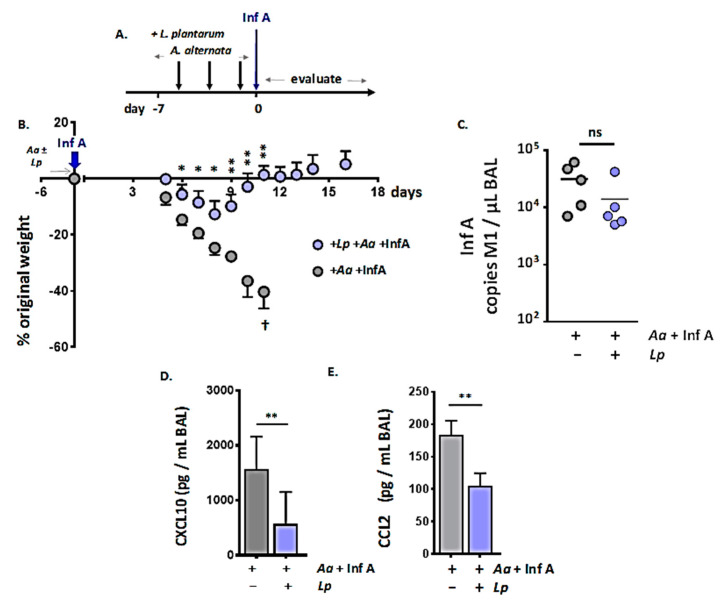
Administration of immunobiotic *Lactobacillus plantarum* directly to the respiratory mucosa protects against the lethal response to Inf A. (**A**) Strategy: repetitive administration of *A. alternata* (days −5, −3 and −1; 2.5 µL per nare, 5 µg per inoculum) immediately preceded (10–15 min) by *L. plantarum* (10^8^ CFU in 50 µL phosphate-buffered saline [PBS] with 0.1% bovine serum albumin [BSA]) or diluent control. On day 0, mice were inoculated with Inf A. (**B**) Administration of *L. plantarum* protects against increased susceptibility to lethal Inf A; *n* = 5 mice per group, * *p* < 0.05; ** *p* < 0.01, ^†^ no survival after this time point in the group indicated. (**C**) Virus titer (copies per µL BAL fluid) on day 8 in mice challenged with *A. alternata* as in Figure 5A both with and without *L. plantarum*; ns, no significant difference. (**D**) CXCL10 (pg/mL BAL fluid) and (**E**) CCL2 (pg/mL BAL fluid); day 8 as per Figure 5A; *n* = 5 per group, ** *p* < 0.01, Mann–Whitney U-test.

As we have demonstrated here, administration of *A. alternata* amplified the Inf A-induced inflammatory response (Figure 2) and targeted AMs (Figure 3). As such, we considered the possibility that *L. plantarum* may serve in an immunomodulatory capacity by limiting inflammatory cytokine production in the respiratory tract and thus minimizing the amplified lethal response. The strategy used to explore this hypothesis is shown in Figure 5A. We administered *L. plantarum* to the respiratory tract on days −5, −3, and −1, immediately prior to the administration of *A. alternata* as shown; these treatments were followed by Inf A on day 0.

As shown in Figure 5B, immunobiotic *L. plantarum* administered directly to the respiratory mucosa limited virus-induced weight loss and also the exaggerated lethal response to Inf A promoted by *A. alternata*. Administration of *L. plantarum* had no impact on virus titer (day 8 of Inf A infection; Figure 5C) or, interestingly, on the amplified loss of AMs secondary to *A. alternata* challenge. However, *L. plantarum* at the respiratory mucosa did result in significant suppression of virus-induced proinflammatory cytokines, notably CXCL10 (Figure 5D) and CCL2 (Figure 5E).

### 3.6. Proinflammatory Components of the A. alternata Filtrate: Serine Proteases, RNases, and Low Molecular Weight Biomolecules

Finally, as a first step toward elucidation of the *A. alternata* components promoting the amplified lethal response to Inf A, we examined several constituents with known proinflammatory and/or cytotoxic activities. Among these, serine proteases are major secretory products and bioactive components of fungal culture filtrates [12,59]. In prior studies, intranasal inoculation of mice with *A. alternata*-derived serine protease (AASP) or the Alp1 protease from *A. fumigatus* induced allergic airway inflammation and bronchial hyperreactivity [11,60]. Fungal proteases typically promote allergic and inflammatory responses via activation of the receptor, proteinase activated receptor 2 (PAR2; [12,13,61,62]), although protease and PAR2-independent allergic and inflammatory responses to *A. alternata* have also been reported [15,63,64].

**Figure 6:**
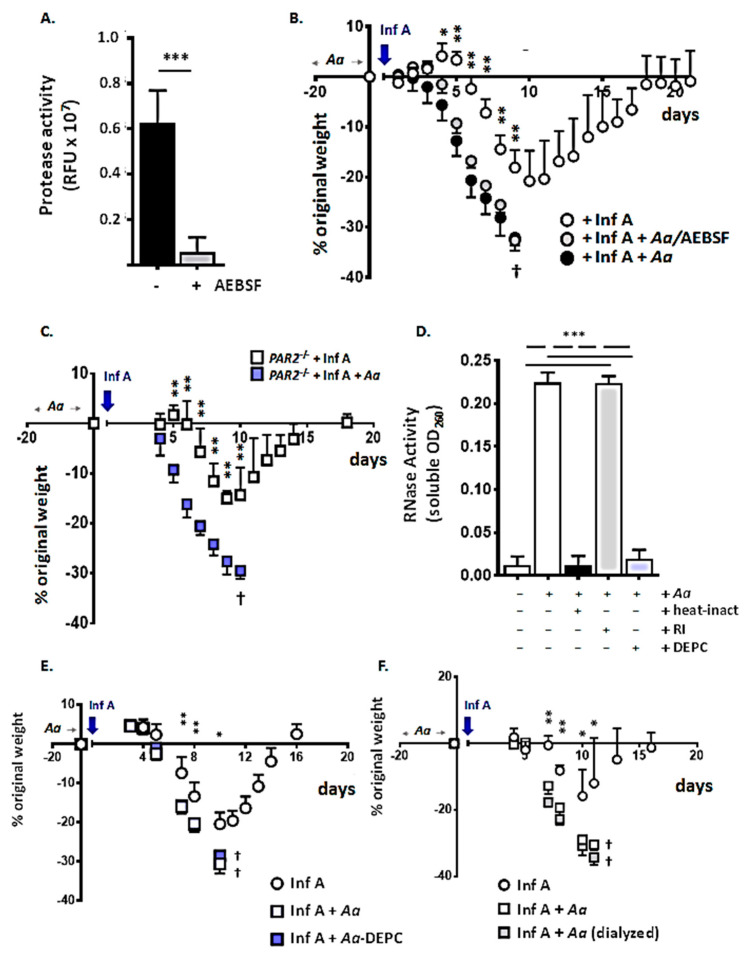
Serine protease activity, RNase activity, and low molecular weight biomolecules are not among the critical factors promoting the amplified lethal response to Inf A. (**A**) Treatment with the irreversible serine protease inhibitor AEBSF reduces the protease activity in filtrates from *A. alternata* by >10-fold; *** *p* < 0.005. (**B**) Proteolytic inactivation of the *A. alternata* filtrate with AEBSF had no impact on its ability to promote weight loss and mortality when administered at 5 µg per inoculum in response to a sublethal infection with Inf A; *n* = 5 mice per group, ** *p* < 0.01, ^†^ no survival after this time point in groups indicated. (**C**) Responses of *Par2* gene-deleted (*Par2*^-/-^) mice to Inf A alone and Inf A after repetitive inoculation with *A. alternata* (as per Figure 1A; 5 µg per inoculum) are similar to those of wild-type mice; *n* = 5 mice per group, ** *p* < 0.01, ^†^ no survival after this time point in the group indicated. (**D**) Heat (95 °C for 10 min) and diethylpyrocarbonate (DEPC), but not human placental ribonuclease inhibitor (RI) inhibited the ribonuclease activity in *A. alternata* filtrates, consistent with the profile of a T2 family enzyme (see Genbank Acc. No. XM_018531878.1); *** *p* < 0.005. (**E**) Ribonucleolytic inactivation of the *A. alternata* filtrate with DEPC has no impact on its capacity to promote weight loss and mortality (5 µg per inoculum) in response to an otherwise sublethal Inf A infection; *n* = 5 mice per group, ** *p* < 0.01, 1-way ANOVA; ^†^ no survival after this time point in groups indicated. (**F**) Dialysis of the *A. alternata* filtrate to remove small biomolecules (<12 kDa) had no impact on the amplified lethal response to Inf A; ** *p* < 0.01, * *p* < 0.05, ^†^ no survival after this time point in the group indicated.

In our earlier study [16], we showed that some (but not all) of the exaggerated lethal response to Inf A was eliminated by heat-inactivation of the *A. alternata* filtrate; this physical maneuver inactivates protease activity, but it is relatively broad and non-specific. As shown in Figure 6A, treatment with the specific and irreversible serine protease inhibitor, AEBSF, resulted in a more than 10-fold loss of proteolytic activity characteristic of *A. alternata* filtrates. However, as shown in Figure 6B, inactivation of serine protease activity had no impact on the amplified lethal response elicited by *A. alternata* filtrates. Similarly, mice devoid of the serine proteinase-activated receptor, PAR2, responded to repetitive exposure to proteolytically-active *A. alternata* filtrates in a manner that was indistinguishable from the wild-type (Figure 6C). These results indicate that *A. alternata* serine proteases do not play a critical role in promoting the amplified lethal response to Inf A infection.

Given the critical role played by endogenous, host-derived proteases in activating Inf A virus viruses and promoting infectivity and inflammation (i.e., the cytokine storm; [65,66]), this finding was quite unexpected. However, while administration of *A. alternata* promoted a lethal response to sublethal Inf A, this treatment had no impact on BAL fluid virus titer (Figure 1D). We also found that the proteolytically-active filtrate was not capable of activating Inf A in tissue culture experiments, as performed effectively by TPCK-trypsin in cultures with target MDCK cells.

We also considered the possibility that ribonucleases (RNases), another heat-susceptible component of the *A. alternata* filtrate, might contribute to the exaggerated responses observed. *A. alternata* synthesizes and secretes a T2 RNase (GenBank Acc. No. XM_018531878.1). Members of the T2 family of RNases have been identified in bacteria, plants, animals, and viruses, and have diverse roles as cytotoxins and immunomodulatory agents [67,68]; most recently, Acquati and colleagues [69] reported that human T2 RNase functions as a tumor suppressor gene. As a group, T2 RNases cleave single-stranded RNA substrates in vitro and are identified by homology within the CAS I and CAS II catalytic domains to the prototype, the RNase T2 originally isolated from the fungal species *Aspergillus oryzae*.

As shown in Figure 6D, the activity profile of the *A. alternata* filtrate is consistent with that anticipated for a T2 RNase. RNase activity was not inhibited by mammalian cellular RNase inhibitor (RI), an intracellular protein that targets RNase A family ribonucleases [70] but was irreversibly inactivated by heating (95 °C for 10 min) and by treatment with diethyl pyro-carbonate (DEPC). However, as shown in Figure 6E, the DEPC-treated, ribonucleolytically inactivated *A. alternata* filtrate was equally effective as its enzymatically active, DEPC-naïve counterpart at eliciting an amplified lethal response to Inf A. These results indicate that the *A. alternata* T2 RNase was also not a critical factor promoting the exaggerated lethal impact of Inf A.

Finally, we considered the possibility that the amplified response to Inf A might be elicited by one or more low molecular weight biochemical components of the *A. alternata* filtrate. Bioactive molecules such as alternariol and alternariol monomethyl ether (MW 258 and 272 g/mol, respectively, reviewed in [71,72,73]) have been evaluated primarily as fungal food contamination. However, as noted above, alternariol has also been characterized as a cytotoxin that targets human and mouse macrophages [42,74,75].

To address this question, *A. alternata* stock filtrates were subjected to extensive dialysis (>2000 volumes) so as to eliminate all molecules smaller than 12 kDa (12,000 g/mol). Nonetheless, as shown in Figure 6F, the dialyzed filtrate was fully capable of eliciting an amplified response to Inf A; no significant differences from the responses to the non-dialyzed filtrate were observed.

These studies effectively exclude the possibility that small molecules contained in the *A. alternata* filtrate play critical roles in promoting increased susceptibility to Inf A. While it remains possible that small fractions of these biomolecules remained attached high-molecular weight carrier proteins and thereby can resist filtration, the fact that extensive dialysis had no impact whatsoever on the *A. alternata*-mediated amplified response to Inf A argues against this possibility.

### 3.7. Discussion

In our previous study, we identified a means to elicit Th2 inflammation in the upper airways of wild-type mice by repetitive administration of *A. alternata*. We showed that this strategy resulted in an eosinophil-independent *increased* susceptibility to the lethal sequelae of Inf A infection [16]. Here, we demonstrate that the exaggerated response promoted by *A. alternata* was not dependent on serine protease activity, ribonuclease activity, or small molecules present in the filtrate. However, repetitive challenge with *A. alternata* did result in elevated levels of proinflammatory cytokines and neutrophil recruitment to the lower airways to levels that exceeded those observed in response to Inf A infection alone. These findings were also associated with a profound loss of AMs and spontaneous accumulation of bacteria in the airways; however, concomitant treatment with ampicillin eliminated spontaneous bacterial growth but had no impact on the exaggerated lethal response. By contrast, administration of immunomodulatory *L. plantarum*, a strategy shown previously to limit virus-induced airway inflammation [28,55] reversed the inflammation and eliminated the exaggerated response to a sublethal inoculum of Inf A.

Among the questions remaining to be answered, we do not have a clear understanding of the molecular mechanisms that promote a lethal response to what would otherwise be a sublethal inoculum of Inf A. In our earlier study [16] we examined the status of the upper airways immediately prior to virus inoculation. We found that repetitive challenge with *A. alternata* resulted in profound recruitment of eosinophils to the nasal passages accompanied by elevated levels of IL-5, IL-13, eotaxin-1, and IgA in nasal wash fluid. Interestingly, and despite their characterized antiviral properties [17,18,19,20], eosinophil recruitment to the upper airways and nasal mucosae provided no protection against the lethal sequelae of the otherwise sublethal dose of Inf A. Likewise, findings presented here indicate that proinflammatory cytokine and leukocyte levels in the lower airways are at baseline (i.e., comparable to naïve mice) at day 6 after the final strict intranasal dose of *A. alternata* in control mice that were not infected with Inf A (Figure 2 and Figure 3). This of course does not preclude the possibility of elevated cytokine and leukocyte levels at day 0. Indeed, a cytokine response detected immediately after administration of *A. alternata* may be among the primary effective targets of *L. plantarum* (Figure 5). Nonetheless, our preliminary findings that suggest that there is little to no leukocyte recruitment to the lower airways at day 0 in mice subjected to repetitive strict intranasal dosing with *A. alternata* alone.

Taken together, these observations may be critical toward our understanding of the approach severe Inf A infections in human subjects, notably in those individuals who are chronically exposed to molds and fungi [76,77,78,79].

## Supplementary Materials

The following are available online at https://www.mdpi.com/1999-4915/12/9/946/s1, Figure S1: Identification of leukocyte subsets by flow cytometry; Figure S2: Microscopic evaluation of lung tissue from mice subjected to repetitive administration of *A. alternata* prior to Inf A infection; Figure S3: Repetitive administration of *A. alternata* has no impact on the inflammatory response to Inf A infection in primary mouse respiratory epithelial cell culture (mTEC).
