# Supplementary material for: Alternaria alternata Accelerates Loss of Alveolar Macrophages and Promotes Lethal Influenza A Infection
Source: Viruses. 2020 Aug 27;12(9):946. doi: 10.3390/v12090946 (PMC7552021; doi:10.3390/v12090946)
Supplement: Supplementary file 1 [file viruses-12-00946-s001.zip › Suppl Figure S1.pdf]

Supplemental Figure S1. Identification of leukocyte subsets by flow cytometry. Single cell suspensions from lung tissue were subjected to flow cytometry to identify **A.** myeloid and **B.** lymphoid cell lineages via strategies as shown.

A.

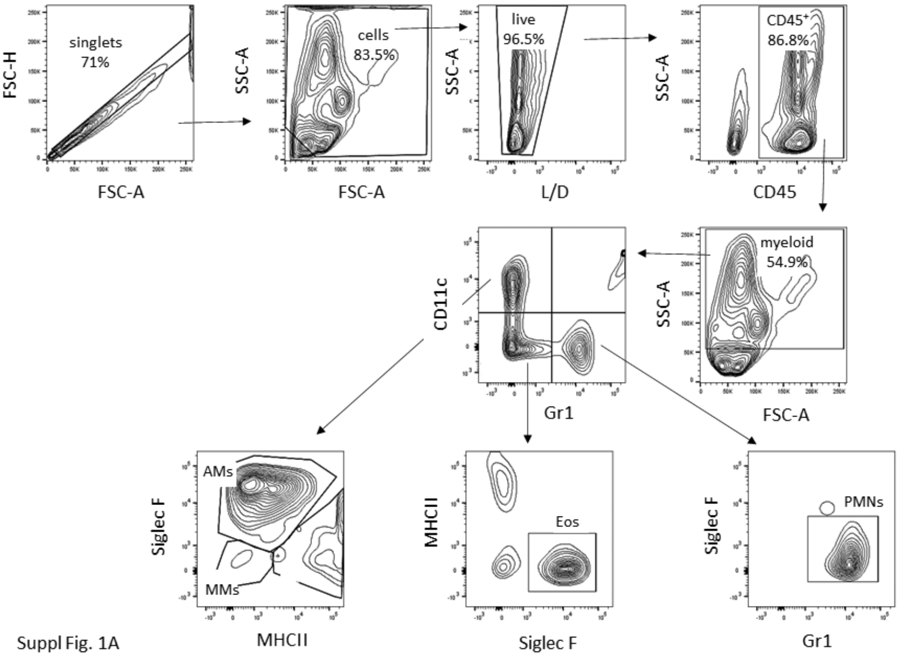

Suppl Fig. 1A

B.

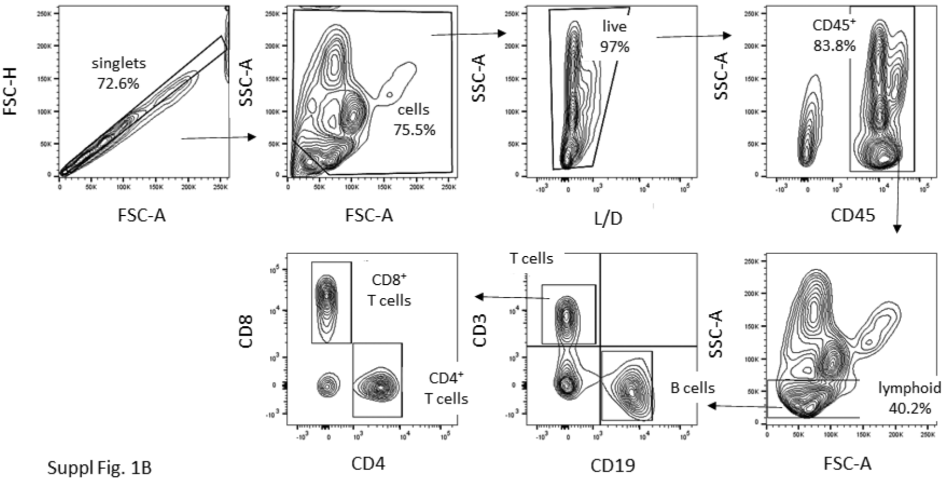

Suppl Fig. 1B
