# Supplementary material for: Alternaria alternata Accelerates Loss of Alveolar Macrophages and Promotes Lethal Influenza A Infection
Source: Viruses. 2020 Aug 27;12(9):946. doi: 10.3390/v12090946 (PMC7552021; doi:10.3390/v12090946)

Supplemental Figure S2. Microscopic evaluation of lung tissue from mice subjected to repetitive administration of *A. alternata* prior to Inf A infection. **A.** H & E staining, original magnification, 10x; area within the box is enlarged in **B.** with arrows highlighting necrotic debris and inflammatory cells infiltrating the bronchi, original magnification 20x. **C.** Detection of Inf A-infected cells with rabbit polyclonal anti-NP (10x); area within box enlarged in **D.**, arrows highlight virus-infected bronchial epithelial cells (64x).

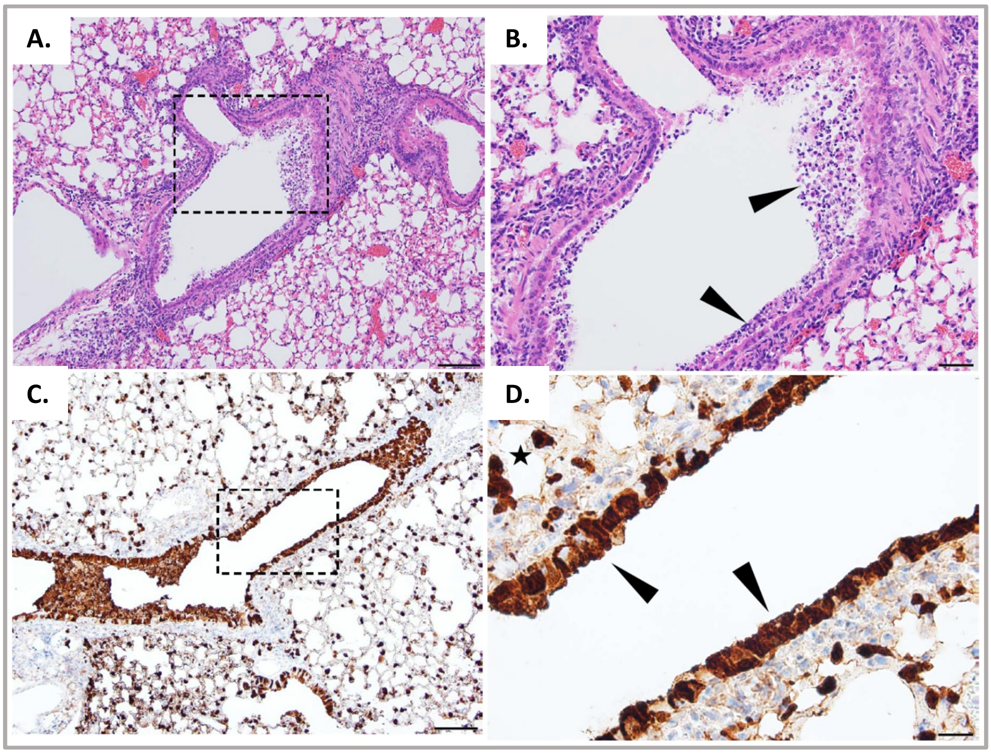

Supplement: Supplementary file 1 [file viruses-12-00946-s001.zip › Suppl Figure S2.pdf]
