# Supplementary material for: Alternaria alternata Accelerates Loss of Alveolar Macrophages and Promotes Lethal Influenza A Infection
Source: Viruses. 2020 Aug 27;12(9):946. doi: 10.3390/v12090946 (PMC7552021; doi:10.3390/v12090946)

Supplemental Figure S3. Repetitive administration of *A. alternata* has no impact on the inflammatory response to Inf A infection in primary mouse respiratory epithelial cell culture (mTEC) A. Virus (Inf A/FM/1/47-MA) detected in cells by qPCR (copies per GAPDH) at day 4 of infection; **B.** TNFα (pg/mL basal medium); **C.** CXCL10 (pg/mL basal medium) **D.** CCL2 (pg/mL basal medium); **E.** IL-6 (pg/mL basal medium), **F.** CXCL1 (pg/mL basal medium).

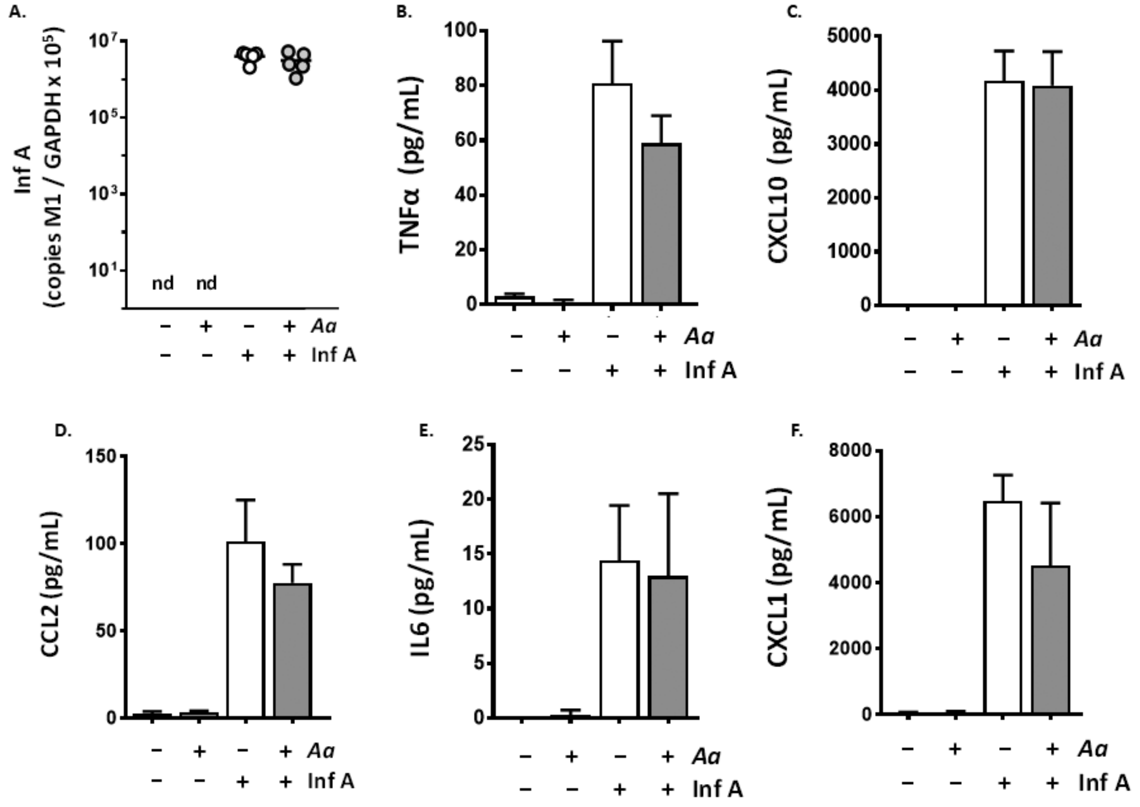

Supplement: Supplementary file 1 [file viruses-12-00946-s001.zip › Suppl Figure S3.pdf]
